# Soil carbon and nitrogen fraction dynamics affected by tillage erosion

**DOI:** 10.1038/s41598-019-53077-6

**Published:** 2019-11-12

**Authors:** Xiao-Jun Nie, He-Bing Zhang, Yan-Yan Su

**Affiliations:** School of Surveying and Land Information Engineering, Henan Polytechnic University, Jiaozuo City, 454000 China

**Keywords:** Ecology, Environmental impact

## Abstract

Understanding the impact of tillage erosion on soil organic carbon (SOC) and nitrogen (N) fractions is essential for targeted soil conservation in mountainous and hilly areas. However, little is known about this issue. In this study, we selected a tillage erosion-dominated hillslope from the Sichuan Basin, China, to determine the effect of tillage erosion on particulate OC (POC), dissolved OC (DOC), light fraction OC (LFOC), ammonium N (NH_4_^+^-N), nitrate N (NO_3_^−^-N) and alkali-hydrolysable N (AN). Additionally, we investigated the microbial activities in relation to soil C and N dynamics, including soil microbial biomass, β-glucosidase and urease activities. Tillage erosion induced serious soil loss in upper slope positions and soil deposition in lower slope positions. The observations of the various labile OC fraction distributions across the hillslope suggest that tillage erosion exerts less impact on DOC and LFOC dynamics but a notable effect on POC. The distribution pattern in total organic carbon under tillage erosion mainly depends on POC redistribution. The POC redistribution is a major factor affecting microbial activities. The AN is more prone to the tillage erosion impact than NH_4_^+^-N and NO_3_^−^-N. Effective soil conservation measures should be taken to weaken the adverse impacts of tillage erosion on POC and AN redistribution in sloping farmlands.

## Introduction

Globally, vast areas of agricultural lands have undergone serious degradation because of soil erosion^1^. It is well known that temperate agricultural soils in mountainous and hilly areas are affected by tillage and water erosion at around the same order of magnitude^2^. On steeply sloping farmlands, soil loss due to intensive tillage can even exceed the loss by water erosion^3–7^. For example, Richter (1999) reported that soil loss due to tillage operations was six times larger than the loss by water erosion^5^. Tillage erosion is tillage-induced lateral movement of soil on sloping fields or refers to the net soil translocation downslope due to tillage operations (i.e., hoeing or plowing). Tillage erosion can trigger severe soil loss at upper slope positions and soil deposition at lower slope positions, often resulting in net soil loss in steeply sloping landscapes^8,9^. The adverse impact of tillage erosion is an increasing threat to sustainable agriculture in mountainous and hilly areas.

In recent years, the impacts of tillage erosion on soil properties have received more attention worldwide. Numerous studies have demonstrated that tillage erosion can lead to increasing within-field variations of soil organic carbon (SOC) and nitrogen (N) stocks, resulting in crop yield reduction^4,10–12^. Additionally, tillage erosion acts as a delivery mechanism for water erosion and thus accelerates soil loss, which is accompanied by SOC depletion through water erosion within the same slope^13^. However, previous studies focus on the tillage erosion impact on total organic carbon (TOC) and total nitrogen (TN), and little is known about the fates of labile soil C and available N fractions under the effects of tillage erosion. Soil labile C and available N parameters such as particulate organic C (POC), dissolved organic C (DOC), light fraction organic C (LFOC), alkali-hydrolysable N (AN), ammonium N (NH_4_^+^-N) and nitrate N (NO_3_^−^-N) are active fractions of soil C and N pools, and they can respond sensitively to soil erosion^14^. Therefore, more studies are needed to clarify how the labile C and available N fractions respond to tillage erosion to take targeted measures of soil conservation in mountainous and hilly areas.

Soil microorganisms are important participants in driving soil C and N cycles. Changes in microbiological indicators such as soil microbial biomass and special enzyme activities (e.g., β-glucosidase and urease) under soil disturbances can apparently affect the biochemical transformations of soil C and N^15,16^. It has been documented that soil erosion may reduce soil microbial biomass and enzyme activities associated with C and N transformations^17–20^. Most of these studies center the impact of water erosion, but only a handful of studies center the impact of tillage erosion on microbial activities^20^. Moreover, what linkages exist between soil C, N fractions and microbial activities under tillage erosion remains unclear.

An artificial radionuclide from atomic weapons testing and emissions from nuclear power stations, ^137^Cs, is a well-established tracer for determining soil redistribution patterns (i.e., soil erosion and deposition) at field scales^21^. In this study, we used the same methodology to investigate tillage erosion-induced soil redistribution on a steep hillslope of the Sichuan Basin, with the aim to 1) examine the differences in the various soil C and N fraction contents and microbial activities between erosional and depositional slope positions and 2) elucidate the effects of tillage erosion on soil C and N dynamics, as well as the relationships between the C and N dynamics and microbial activities under tillage erosion.

## Results

### Soil redistribution

**Figure 1:**
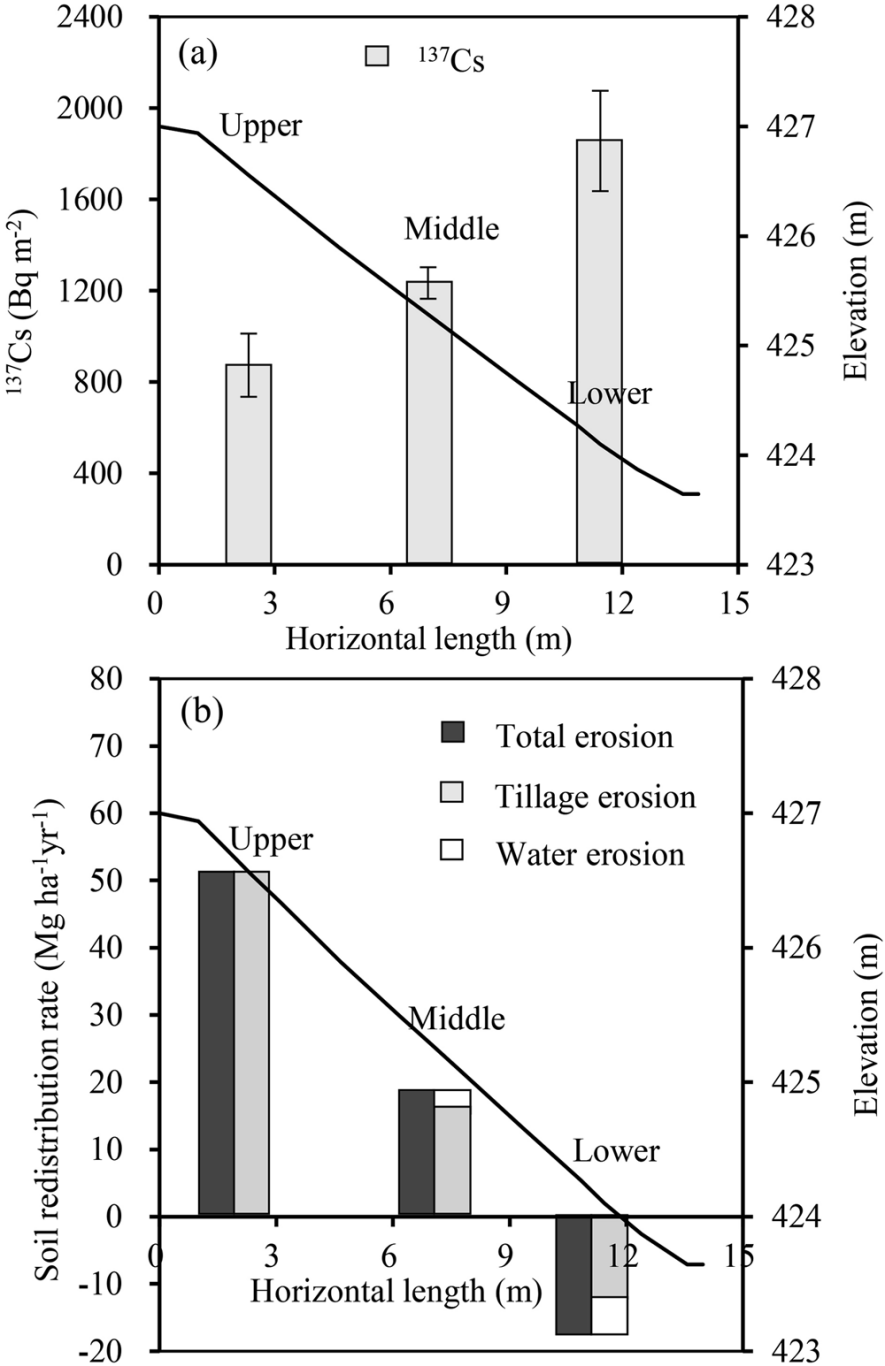
^137^Cs variation (**a**) and erosion-induced soil redistribution (**b**) along the hillslope. The slope profile (metres above sea level) is indicated by the black line. In figure a, the error bars are the standard error of the mean and different letters for ^137^Cs indicate the significant differences between slope positions. In figure b, the positive and negetive results indicate erosion and deposition, respectively.

Figure 1 displays soil redistribution patterns in different positions on the hillslope. ^137^Cs inventories increased from upper slope to lower slope, with significant differences between slope positions (*Ps* < 0.05; Fig. 1a). Compared with a local reference inventory (1463 Bq m^−2^), the ^137^Cs inventories from the upper (874 ± 138 Bq m^−2^) and middle (1234 ± 69 Bq m^−2^) slope positions were smaller, while the ^137^Cs inventories (1856 ± 220 Bq m^−2^) from the lower slope were greater. The results showed the upper and middle slope positions were erosional and the lower slope was depositional. Total erosion rates were estimated at 52 Mg ha^−1^ yr^−1^ for upper slope positions (0–4.8 m of slope length) and 19 Mg ha^−1^ yr^−1^ for middle slope positions (Fig. 1b), indicating that the upper and middle portions of the hillslope were strongly erosional and weakly erosional, respectively. Total soil deposition was estimated at 17 Mg ha^−1^ yr^−1^ in the lower slope positions (Fig. 1b). Overall, the hillslope exhibited a net soil loss. The tillage erosion rates accounted for 100% and 88% of the total erosion rates in the upper and middle slope positions, respectively. In the lower slope positions, soil deposition by tillage erosion had a 69% contribution to the total soil deposition. Thus, tillage erosion was a predominant erosion process on the hillslope.

**Figure 2:**
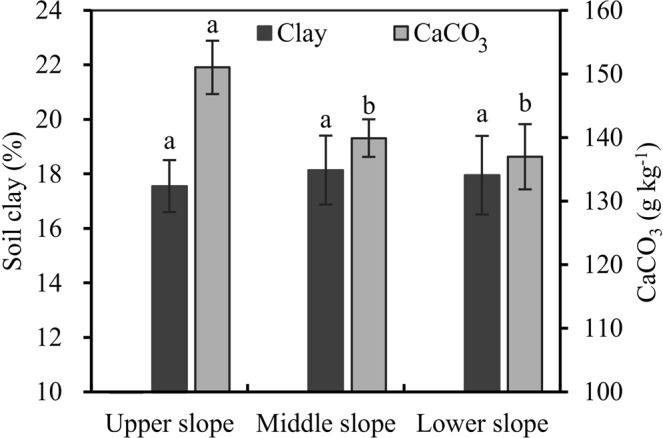
Soil clay and CaCO_3_ contents along the hillslope. The error bars are the standard error of the mean. Different lowercase letters for each indicator show the significant differences between slope positions.

Similar contents of soil clay across the hillslope were observed (Fig. 2), suggesting nonselective transport of fine soil particles. This result also confirmed that tillage erosion was a predominant erosion process on the hillslope. In addition, higher CaCO_3_ contents in the upper slope than in other slope positions were also detected (Fig. 2), which indicated the incorporation of CaCO_3_-rich bedrock fragments into tillage layer after the topsoil was severely eroded by intensive tillage in the upper slope positions. Thus, the CaCO_3_ data further confirmed the most severe soil loss from the upper slope positions on the hillslope.

### Distribution of various soil C and N fractions

The contents of the selected soil C and N fractions in different positions on the hillslope are displayed in Table 1. The TOC contents increased down the hillslope and showed a significant difference between strongly eroded upper slope positions and depositional lower slope positions (*P* < 0.05). In terms of various organic C fractions (i.e., POC, LFOC and DOC), only the POC contents varied significantly with slope position and exhibited the lowest values in the strongly eroded upper slopes and the highest values in the weakly eroded middle slopes (*P* < 0.05). Significant within-field variations in TN, NH_4_^+^-N, NO_3_^−^-N and AN contents were also observed on the hillslope (*Ps* < 0.05). Lower values of TN, NH_4_^+^-N and NO_3_^−^-N occurred in only the strongly eroded upper slopes, while lower AN values occurred in both the strongly eroded upper slopes and weakly eroded middle slopes. These results indicate that soil redistribution by tillage erosion exerted an important impact on the distributions of TOC, POC, TN, NH_4_^+^-N, NO_3_^−^-N and AN on the hillslope.

**Table 1:** Soil C, N and microbial activity parameters from different hillslope positions.

	Upper	Middle	Lower	*P*-value
TOC (g kg^−1^)	4.53 ± 0.79b	5.82 ± 1.10ab	7.27 ± 0.66a	0.02
POC (g kg^−1^)	0.55 ± 0.23c	1.79 ± 0.09a	1.10 ± 0.16b	0.01
LFOC (mg kg^−1^)	292.50 ± 59.09a	197.50 ± 40.31a	240.00 ± 62.18a	0.19
DOC (mg kg^−1^)	113.81 ± 16.33a	93.09 ± 18.20a	85.31 ± 26.24a	0.15
TN (g kg^−1^)	0.88 ± 0.06b	0.98 ± 0.11ab	1.07 ± 0.05a	0.03
NH_4_^+^-N (mg kg^−1^)	5.58 ± 0.40c	7.46 ± 1.20a	6.86 ± 0.42ab	0.02
NO_3_^−^-N (mg kg^−1^)	30.13 ± 2.17b	32.15 ± 1.98ab	38.18 ± 5.61a	0.03
AN (mg kg^−1^)	41.56 ± 2.80bc	41.78 ± 2.42b	50.71 ± 2.90a	0.04

TOC, total organic carbon; POC, particulate organic carbon from coarse soil particles (0.053–2 mm); LFOC, light fraction organic carbon; DOC, dissolved organic carbon; TN, total nitrogen; NH_4_^+^-N, ammonium nitrogen; NO_3_^−^-N, nitrate nitrogen; AN, alkali-hydrolysable nitrogen. Values (mean ± standard deviation) with different letters in a row indicate significant differences between slope positions. Differences among the three slope positions at *P* < 0.05 were considered to be statistically significant.

### Microbial activities related to soil C and N dynamics

**Figure 3:**
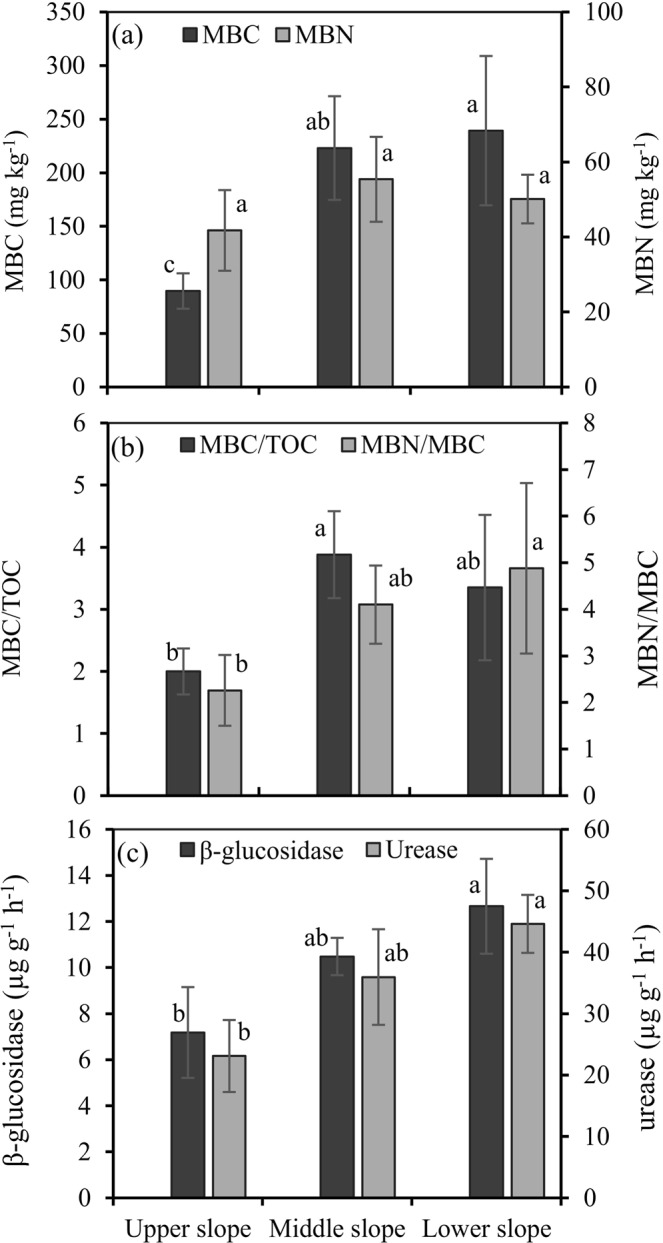
Distribution of microbial activities along the hillslope. MBC, microbial biomass carbon; MBN, microbial biomass nitrogen; MBC/TOC, the ratio of MBC to total organic carbon; MBC/MBN, the ratio of MBC to MBN. The error bars are the standard error of the mean. Different lowercase letters for each indicator show the significant differences between slope positions.

The microbial biomass C (MBC) contents from strongly eroded upper slopes were significantly lower than those from weakly eroded middle slopes and depositional lower slopes (*P* < 0.05), but the microbial biomass N (MBN) contents were similar between the three slope positions (*P* > 0.05) (Fig. 3a). Significantly lower values were observed for the MBC/TOC ratio in the strongly eroded upper slopes compared with the weakly eroded middle slopes (*P* < 0.05) and for the MBC/MBN ratio in the strongly eroded upper slopes compared with the depositional lower slopes (*P* < 0.05) (Fig. 3b). For β-glucosidase and urease, significantly lower activities occurred in the strongly eroded upper slopes compared with the depositional lower slopes (*Ps* < 0.05) (Fig. 3c). A Pearson correlation analysis indicated that the contents of TOC, POC and TN were positively correlated with the MBC contents in the hillslope landscape (Table 2). Positive correlations were also detected between POC, NH_4_^+^-N and MBC/TOC, between TOC and β-glucosidase activity, and between NH_4_^+^-N, AN and urease activity. In addition, there were significant correlations among microbial activity parameters (Table 2). In general, the present findings confirmed an apparent impact of tillage erosion-induced soil redistribution on microbial activities associated with soil C and N dynamics on the hillslope.

**Table 2:** Correlations between soil C, N and microbial activity parameters on the hillslope.

	TOC	POC	TN	NH_4_^+^-N	NO_3_^−^-N	AN	MBC/MBN	β-glucosidase	Urease
MBC	0.65^*^	0.66^*^	0.74^**^	0.56	0.52	0.50	0.87^***^	0.72^**^	0.70^*^
MBC/TOC	0.23	0.74^**^	0.53	0.61^*^	0.21	0.13	0.89^***^	0.56	0.41
β-glucosidase	0.63^*^	0.40	0.65^*^	0.35	0.59^*^	0.52	0.61^*^		
Urease	0.85^***^	0.44	0.50	0.60^*^	0.70^*^	0.78^**^	0.53		

^*^, ^**^, ^***^Significant at the 0.05, 0.01 and 0.001 probability levels, respectively.

## Discussion

Slope degree and slope length are two key topographic factors that impact the tillage erosion rate on sloping farmlands^22,23^. On steep hillslopes with short slope lengths, tillage erosion has been shown to be a major soil redistribution process^2,4,5^. Our study confirmed a similar result. The estimated tillage erosion rate up to 52 Mg ha^−1^ yr^−1^ for upper slope positions can be attributed to tillage erosion-induced net soil output here. Serious soil loss by tillage erosion at the upper slope positions of some steep hillslopes in this study area has been reported to induce the incorporation of CaCO_3_-rich bedrock fragments into tillage layer and even induce the exposure of bedrock^24^. Larger values than our reported values have also been reported by previous studies. For examples, Zhang *et al*. (2004) reported that tillage erosion rates for 12–15° slopes ranged from 70 to 85 t ha^−1^ yr^−1^ in the Sichuan Basin, China^22^. Thapa *et al*. (1999) reported that tillage erosion rate was up to 456 t ha^−1^ yr^−1^ for a 15° hillslope in Claveria, Philippines^25^. In addition, we found that soil erosion rates in the upper and middle slope positions exceed the soil deposition rate in the lower slope positions, exhibiting a net soil loss on the whole hillslope. It is attributed to considerable portions of soil being transported out of the bottom boundary of the hillslope under tillage and water erosion. In hilly areas of the Sichuan Basin, thick sediments that originate from sloping farmlands can be widely observed in drainage ditches.

The results of the present study indicated different distribution patterns in soil C parameters on the tillage erosion-dominated hillslope. Tillage erosion can result in serious TOC depletion in the upper positions of steep hillslopes and obvious TOC enrichment in lower slope positions, thereby increasing the within-field variation in TOC contents^4^. Our results agree with those of previous studies. For the observed labile organic C fractions (POC, DOC and LFOC), only POC showed a similar distribution pattern to the TOC between the strongly eroded upper slopes and depositional lower slopes. This finding suggests that POC dynamics were closely associated with soil redistribution by tillage erosion. POC mainly lies in soil macroaggregates^26^. Previous studies reported that POC redistribution was attributed more to water erosion in sloping farmlands because shear forces of slope runoff can disrupt soil macroaggregates and then lead to POC exposure and resultant POC depletion^27,28^. Our result differs from previous studies. This is because soil macroaggregates can be easily transferred downslope during the process of tillage erosion after soil clods are cut into macroaggregates and microaggregates under tillage operations^8,29^. In the present study, we also found that the POC/TOC values ranged between 8–44% compared with the values of DOC/TOC and LFOC/TOC ranging between 1–8% (Table 1). This indicated that POC was a major organic C fraction in the studied soil landscape. In addition, significantly lower POC contents in depositional lower slopes were observed compared to those in weakly eroded middle slopes, which is likely because a portion of the eroded soils from the middle slopes are directly transported out of the bottom boundary of the hillslope under tillage erosion. Overall, our findings provide insights into SOC redistribution induced by tillage erosion, i.e., the TOC distribution pattern under tillage erosion mainly originates from the POC redistribution contribution.

Unlike the POC, the DOC and LFOC did not exhibit evident depletions in the erosional sites (i.e., upper and middle slope positions) compared with the depositional sites (lower slope positions), which suggests that tillage erosion-dominated soil redistribution exerts less impact on the DOC and LFOC fractions. Previous studies indicated runoff transport-related mineralization of DOC and preferential removal of fine soil particle-bound LFOC in the process of water erosion^30–32^. In the present study, tillage erosion was confirmed to be a predominant erosion process. Moreover, soil clay data showed that tillage erosion didn’t selectively transport fine soil particles. Thus, the dynamics of DOC and LFOC were less impacted by tillage erosion.

On the hillslope, various soil N parameters exhibited similar distribution patterns and a pattern similar to that of TOC distribution. Previous studies have documented that TN distribution under tillage erosion is similar to TOC distribution^11,33,34^. Our results further indicate that soil loss by tillage erosion can also induce obvious depletions in NH_4_^+^-N, NO_3_^−^-N and AN, and soil deposition by tillage erosion can induce enrichments in these parameters. In addition, only the AN contents were observed to be significantly lower in weak erosion positions than those in depositional positions of the hillslope, which suggests that the AN fraction is more prone to the impact of soil loss by tillage erosion than the NH_4_^+^-N and NO_3_^−^-N fractions.

The results of this study also highlighted the impact of tillage erosion on microbial activities. The observations of a similar pattern in MBC to those of TOC and TN distributions and the significant correlations among TOC, TN and MBC suggest that under tillage erosion, the change in soil microbial biomass is closely associated with soil TOC and TN redistribution. The soil MBC/MBN ratios averaged 3.8 ± 1.6 on the hillslope (Fig. 3), indicating that the soil microbial biomass is mainly contributed by the bacterial community^35^. The activities of β-glucosidase and urease were positively correlated with the MBC contents, which suggests that in the tillage erosion process, the two enzymes participating in microbial decomposition of organic C and N substrates mainly originate from the bacterial community secretion. The MBC/TOC ratio is a more useful assessment indicator that reflects soil microbial activity than either MBC or TOC^36^. Our observations of a significantly positive correlation between the MBC/TOC and POC contents but no significant correlation between the ratio and TOC suggest that POC redistribution under tillage erosion is a major factor affecting soil microbial activities. No significant correlation between TN content and urease activity was observed, indicating that tillage erosion adversely affects the involvement of urease in the N cycle. Additionally, significant positive correlations between NH_4_^+^-N, AN and urease activity were observed, which is ascribed to similar impacts of tillage erosion on the three parameters.

## Conclusions

The results of this study provided new insights into the effects of tillage erosion on soil C and N dynamics in mountainous and hilly areas. Tillage erosion exerts an apparent impact on POC distribution but less impact on the distribution DOC and LFOC fractions in steeply sloping farmlands. The TOC distribution under tillage erosion mainly depends on the POC fraction redistribution. The AN fraction is more prone to the soil loss impact of tillage erosion than the NH_4_^+^-N and NO_3_^−^-N fractions. The POC redistribution is a major factor affecting microbial activities. The negative influences of tillage erosion on POC and AN dynamics in steeply sloping farmlands should be addressed.

## Methods

### Study area

The experiment was conducted in Jianyang County of the Sichuan Basin, southwestern China (30°04′-30°39′N, 104°11′-104°53′E). The study area is one of the most important grain-growing areas of the Sichuan Basin, with crop rotations involving wheat (*Triticum aestivum* L.) and maize (*Zea mays* L.)/sweet potato (*Ipomoea batatas*). This area is typical of the hilly areas of Sichuan (400–587 m height above sea level), and the site has a humid subtropical climate with a mean annual temperature of 17 °C and a mean annual rainfall of 872 mm, 90% of which occurs between May and October. The local soils derived from CaCO_3_-rich purple mudstone in the Jurassic Age were classified as Orthic Regosols^37^. In the soils, illite and montmorillonite are the dominant clay minerals. Consequently, the soil is vulnerable to water erosion. In order to solve the problem of water erosion, most of long hillslopes have been dissected into slope segments in this study area. In long-term agricultural practices, downslope-tillage (tools, hoes; frequency, 2–3 times per year) is a predominant tillage method on sloping farmlands, which triggers severe tillage erosion, especially in steep sloping farmlands. Intense water and tillage erosion led to shallow depths in the soil profile, generally ranging from 0.2 to 0.5 m.

### Soil sampling

Soil sampling was carried out on a representative steep hillslope (slope degree, 13.5°; slope length, 14.4 m; slope width, 80 m) during September 2016. In the hillslope, the topsoil physicochemical properties were as follows: sand 22%, silt 60%, clay 18%, bulk density 1.4 g cm^−3^, soil organic matter 10.1 g kg^−1^, pH value 8.3, and CaCO_3_ 143 g kg^−1^. Soil samples were taken along three parallel downslope transects that were perpendicular to the hillslope contour line. The three parallel transect lines were each separated by 25 metres. For each downslope transect, the sampling points were set up at 0.5, 2.5, 4.5, 5.3, 7.3, 9.3, 10, 12 and 14 m of slope length. There were 9 sampling points each in the upper (0–4.8 m of slope length), middle (4.8–9.6 m) and lower (9.6–14.4 m) slope positions. For each sampling point, two replicated cores (8-cm diameter) were collected to the bedrock. The cores were divided into two subsamples, i.e., one for the tillage layer (0–20 cm) and the other below the tillage layer (20 cm to bedrock). The two replicated subsamples for each sampling layer were bulked to make a composite sample and then immediately stored in plastic bags at 4 °C.

### Laboratory analysis

The composite samples were air-dried before being crushed and sieved (<2 mm) to remove visible gravels and organic residues. The air-dried subsamples from the tillage layer and below the tillage layer were analysed for ^137^Cs. The subsamples from the tillage layer were also analysed for soil particle size fractions, pH, bulk density, TOC, DOC, POC, LFOC, MBC, TN, NH_4_^+^-N, NO_3_^−^-N, MBN, β-glucosidase and urease.

Soil samples used for ^137^Cs determination were packed into plastic (polyvinyl chloride [PVC]) beakers with a volume of 320 cm^3^, and the activity (mass basis, Bq kg^−1^) of ^137^Cs was measured using a hyperpure lithium-drifted germanium detector (HpC - 40% efficiency) coupled with a Nuclear Data 6700 multichannel g-ray spectrophotometer (Ortec, USA). The ^137^Cs activity was detected at 662 KeV, and the count time for each sample ranged from 40,000 to 60,000 s. The ^137^Cs inventory (Bq m^−2^) was obtained using the total net weight (oven-dried soil mass) of the bulked core sample and the cross-sectional area of the sampling device.

Soil particle size fractions were determined with the pipette method^38^. A soil-to-water ratio of 1:2.5 (*w*/*v*) was adopted in measuring the soil pH with a digital pH metre (Metrohm 702, Switzerland). Soil bulk density was determined using net oven-dried weight and sample volume. The TOC was measured by dry combustion with a TOC analyser (vario Elemental, Germany). TN was determined by the classical Kjeldahl digestion method^39^. The DOC was determined in an aqueous extract (*w*/*v*, fresh soil-to-water of 1:10) using a TOC analyser (vario Elemental, Germany). Coarse soil particles (0.053–2 mm) were selected to determine the POC concentration using the method described by Cambardella and Elliot^40^. The LFOC determinations followed the method described by Gregorich and Ellert^41^. The alkali solution diffusion method was used to determine the AN concentration^38^. Fresh soil samples were extracted with 0.01 M CaCl_2_ by shaking for 1 h at a soil-to-solution ratio of 1:10 and measured with a continuous flowing analyser (AA3, Seal, Germany) to determine the NH_4_^+^-N and NO_3_^−^-N concentrations. The MBC and MBN were determined using the chloroform fumigation-extraction method, using a *K*_*C*_ factor of 0.38 and a *K*_*N*_ factor of 0.45^42^.

### Soil redistribution rate

The total soil erosion rate at an erosional point and deposition rate at a depositional point were calculated using the simplified mass-balance model and the proportional model^43,44^, which are expressed as (Eqs 1 and 2):

1 $${E}_{t}=\frac{10{\rm{dB}}}{P}[1-{(\frac{C{s}_{i}}{C{s}_{r}})}^{1/(t-1963)}]$$

2 $${D}_{t}=10\times \frac{C{s}_{i}-C{s}_{r}}{{W}_{i}(t-1954)}$$

where *E*_*t*_ and *D*_*t*_ are the total erosion and deposition rates (Mg ha^−1^ yr^−1^), respectively; *d* is the sampling depth (m); *B* is the soil bulk density for the tillage layer (Mg m^−3^); *Cs*_*i*_ is the ^137^Cs inventory at the sampling point (Bq m^−2^); *Cs*_*r*_ is a local ^137^Cs reference inventory (1463 Bq m^−2^)^45^; *W*_*i*_ is the ^137^Cs activity at the sampling point (Bq kg^−1^); *P* is the particle-size correction factor (assumed to be *P* = 1); *t* is the sampling year; 1963 is the peak year of worldwide ^137^Cs fallout; and 1954 is the starting year of worldwide ^137^Cs fallout. The total erosion rate in an erosional slope segment was calculated based on the mean erosion rate of individual sampling points. The total deposition rate in a depositional slope segment was calculated based on the mean deposition rate of individual sampling points.

For a given slope segment, the tillage erosion rate is expressed as follows (Eqs 3–5)^22^:

3 $${T}_{i}=10({Q}_{s,out}-{Q}_{s,in})/{L}_{i}$$

4 $${Q}_{s,out}={D}_{t,i}\cdot {\rho }_{b,i}\cdot ({k}_{1}+{k}_{2}{S}_{i})$$

5 $${Q}_{s,in}={D}_{t,i-1}\cdot {\rho }_{b,i-1}\cdot ({k}_{1}+{k}_{2}{S}_{i-1})$$

where *T*_*i*_ is the soil redistribution rate by tillage erosion for a given slope segment (Mg ha^−1^ yr^−1^), and positive and negative *T*_*i*_ values indicate erosion and deposition, respectively; *Q*_*s,out*_ and *Q*_*s,in*_ are the downslope soil fluxes caused by tillage (kg m^−1^ yr^−1^); *L*_*i*_ is the slope length of the *i*^th^ segment (m); *ρ*_*b,i*_ and *ρ*_*b,i−*1_ are the bulk densities of the tillage layer (*k*_*1*_ and *k*_2_, 0.1066 and 0.4902 kg m^−3^, respectively); *D*_*t,i*_ and *D*_*t,i−*1_ are the tillage depths (m); *k*_*1*_ and *k*_2_ are the soil displacement distance coefficients (m yr^−1^); and *S*_*i*_ and *S*_*i−1*_ are the slope gradients of the *i*^th^ and (*i-1)*^th^ segments (m m^−1^), respectively. For an erosional slope segment, the water erosion rate was estimated using the total erosion rate minus soil redistribution rate by tillage erosion. For a depositional slope segment, the soil deposition rate by water erosion was estimated using the total deposition rate minus soil redistribution rate by tillage erosion.

### Data analysis

Statistical analyses were conducted using SPSS 19.0 software (SPSS, Chicago, IL, USA). The Two-Independent Sample Nonparametric Tests were used to compare the differences in various C and N fraction contents and microbial activities between slope positions. The K-Independent Sample Nonparametric Tests were used to compare the differences in these parameters among slope positions on the hillslope. Differences between groups at *P* < 0.05 were considered to be statistically significant.
